# Modeling and live imaging of mechanical instabilities in the zebrafish aorta during hematopoiesis

**DOI:** 10.1038/s41598-021-88667-w

**Published:** 2021-04-29

**Authors:** Dmitrii Chalin, Charlotte Bureau, Andrea Parmeggiani, Sergei Rochal, Karima Kissa, Ivan Golushko

**Affiliations:** 1 Research and Education Center “Materials”, Don State Technical University, 1 Gagarin Square, Rostov-on-Don, 344000 Russia; 2 LPHI, University of Montpellier, CNRS, INSERM, Montpellier, France; 3 Laboratoire Charles Coulomb, University of Montpellier, CNRS, Montpellier, France; 4 Faculty of Physics, Southern Federal University, Zorge 5, Rostov-on-Don, 344090 Russian Federation

**Keywords:** Biological physics, Tissues, Haematopoiesis, Morphogenesis

## Abstract

All blood cells originate from hematopoietic stem/progenitor cells (HSPCs). HSPCs are formed from endothelial cells (ECs) of the dorsal aorta (DA), via endothelial-to-hematopoietic transition (EHT). The zebrafish is a primary model organism to study the process in vivo. While the role of mechanical stress in controlling gene expression promoting cell differentiation is actively investigated, mechanisms driving shape changes of the DA and individual ECs remain poorly understood. We address this problem by developing a new DA micromechanical model and applying it to experimental data on zebrafish morphogenesis. The model considers the DA as an isotropic tubular membrane subjected to hydrostatic blood pressure and axial stress. The DA evolution is described as a movement in the dimensionless controlling parameters space: normalized hydrostatic pressure and axial stress. We argue that HSPC production is accompanied by two mechanical instabilities arising in the system due to the plane stress in the DA walls and show how a complex interplay between mechanical forces in the system drives the emerging morphological changes.

## Introduction

Mechanical forces arising within tissues is an integral part of any morphogenic process^1–3^. Individual cells adjust gene expression based on external mechanical forces through mechanosensing and mechanotransduction. Thus, they can actively react to external mechanical cues by changing their size, shape, position, and ultimately fate^4–6^. Individual cells can generate force by remodeling their actomyosin networks and transmit it through adhesive complexes within tissues^7^. This complex interplay between cell differentiation, force sensing and generation on the cellular level and force transmission, large-scale shape changes, and morphogens gradients on the tissue level orchestrate morphogenesis and patterning of tissues^2,8–10^.

The major challenge in studying morphogenesis and particularly the role of mechanical constraints is to monitor mechanical forces and shape changes in vivo and relate them to biochemical processes. Microscopy is a leading force in morphogenetic research that helps interlinking events on tissue and cellular levels. In the recent decade, the evolution of image acquisition tools has made possible 4D monitoring of tissue development down to individual cellular borders with high temporal resolution^9–13^. Simultaneously, new invasive experimental techniques such as laser nano-dissection and micropipette aspiration have provided a novel insight into the mechanical and adhesive properties of cells and tissues^14–16^. Rationalizing emerging massive amounts of new experimental data on quantitative and even qualitative level is often impossible without appropriate mathematical models.

The classical theory of elasticity successfully describes shapes of metallic rods, plates, and pipes under mechanical constraints. Once subjected to critical stress, such a system develops instabilities that lead to shape changes breaking its symmetry^2,17–20^. Regardless of the underlying gene machinery, the resulting shape and distribution of stress in a biological tissue often can be considered as a result of a system trying to minimize its elastic energy at every step of the morphogenic process^9,21,22^. Generalizations of linear elasticity in combination with novel experimental methods have been extremely helpful for morphogenetic studies. It has been shown that shape genesis in many living systems is not specified directly through a genetically determined program but instead emerges in a sequence of morphological changes driven by mechanical instabilities. Nature uses spontaneous symmetry breaking to facet viral capsids^23,24^ and shape cell membranes^25–27^, form villi and pits in the intestine^22,28^, produce complex shapes of flowers^29,30^, fruits and vegetables^31,32^. All these examples can be qualified as some sort of mechanical buckling-like instabilities relevant in the current research context; for a broader review on criticality in living systems, see Ref.^33^.

Here, we study the role of mechanical stress in the production of hematopoietic stem/progenitor cells (HSPCs) in the embryonic dorsal aorta (DA) of zebrafish. Conversion of endothelial cells (ECs) comprising the aorta to HSPCs was discovered in zebrafish in 2010 and called endothelial-to-hematopoietic transition (EHT)^34^. Although DA is a relatively compact system with a radius of a few tens of micrometers, it demonstrates complex behavior. In less than 50 h aorta undergoes an almost two-fold variation of its cross-section area and develops a sinusoidal deformational pattern along its main axis. Without losing integrity, DA extrudes individual cells that become HSPCs. After over a hundred years of hematopoiesis research, an embryonic aorta is considered the primary provider of HSPC with long-term replenishment potential, which are precursors of all blood cell lineages^35^. Moreover, the EHT mechanism is highly similar among all vertebrates^13^, which draws much attention to the topic and makes it particularly relevant for regenerative medicine.

Comparing the development and functioning of mutant or drug-treated specimens with that of wild-type ones is a common approach to deconstruct and study complex phenomena in living systems starting from vesicular transport^36,37^ to development of vascular diseases^38,39^, eye pathologies^40,41^ and cancer^42^. By adopting this methodology, it has been shown that successful realization of the hematopoiesis via EHT depends on many factors, such as hypoxia^43^, blood flow^12,34,44^, actomyosin activity^11,34,45^. A large body of works focuses on molecular machinery underlying various cellular processes driving hematopoiesis. For example, usually, the blood flow-induced shear stress is considered only as a signal for the cells of hemogenic endothelium (endothelium, which can produce HSPCs) to start hematopoiesis by adjusting their gene expression^13,34,46^. In general, cells can sense the blood flow via structural changes of the cytoskeleton^47^, cation channels^48^, and caveolae^49^.

Our work complements these studies by putting spotlight on tissue mechanics. We study the role of mechanical stress arising due to activities of individual cells, blood flow, and surrounding tissues in aorta development and HSPCs production, thus bridging gene expression, the behavior of individual cells, and observed morphology, including modulation of aorta diameter. Our work relies on theoretical modeling and 4D fluorescent microscopy data both obtained by us and presented in the literature.

To our knowledge, a comprehensive mathematical description of the system on the tissue level is still missing. Here we develop a new analytical model based on such classical methods and approaches as the continuous theory of thin elastic shells and the phase transition theory. The proposed model considers DA as a cylindrical shell of isotropic elastic material subjected to the pressure difference and external elastic forces arising from its interaction with a surrounding tissue matrix. We show that the interplay between hydrostatic pressure and axial compressing force exerted by surrounding tissues defines the changing shape of the dorsal aorta and influences cell extrusion. Within the developed theoretical framework, we analyze new microscopy data on morphogenesis in wild-type zebrafish embryos, embryos with reduced or absent blood flow (silent-heart mutants), and ones with disrupted actomyosin machinery. We show that mechanical forces not only serve as signals promoting and synchronizing EHT but also assist the process by activating shape instabilities directly.

## Experimental background

Zebrafish is a primary source of knowledge in HSPC development studies due to its advantages in fast reproduction cycle and high transparency in the optical spectrum^50^. Despite the tremendous popularity of this model organism, data with high temporal and spatial resolution on its dorsal aorta morphogenesis has become available only in the second decade of the twenty-first century^34^. Below we briefly review the known data on the development of the zebrafish dorsal aorta during the hematopoiesis (when the endothelial-to-hematopoietic transition takes place).

**Figure 1:**
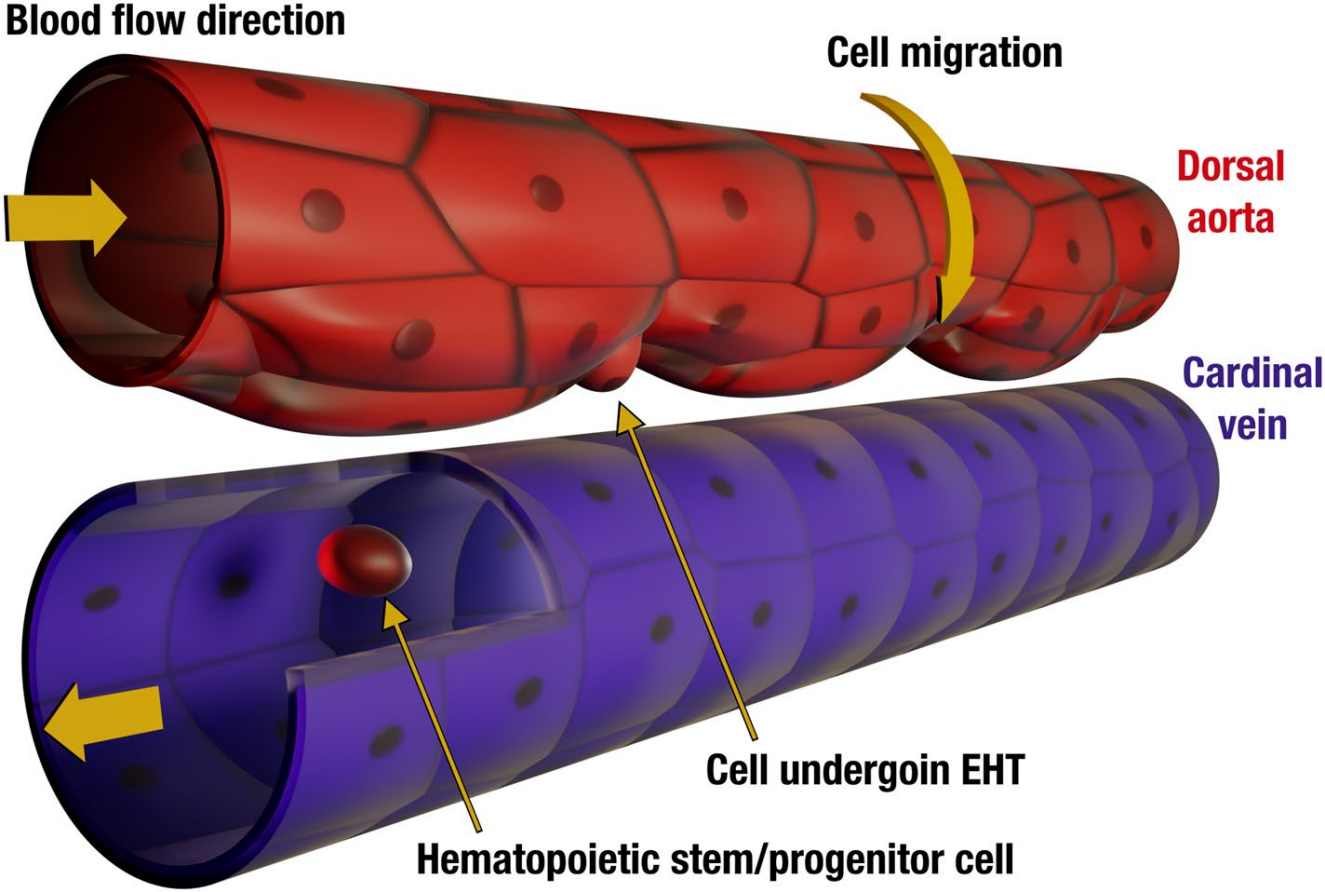
Illustration of zebrafish embryonic dorsal aorta and cardinal vein at 50 hpf. HSPC production is accompanied by a collective cell migration toward the ventral part of the DA and an emergence of the periodic pattern of alternating thinner and thicker regions with a well-defined wave vector. Cells that undergo endothelial-to-hematopoietic transition leave the aorta and enter the cardinal vein becoming HSPCs (shown through a longitudinal section in the cardinal vein). Horizontal yellow arrows show blood flow direction.

During the time period relevant for EHT (~ 24–72 h post-fertilization)^12,13,34^, zebrafish DA is a long tube formed by a monolayer of flat endothelial cells (so-called squamous epithelium) (Fig. 1). The embryonic dorsal aorta is filled with fluid and immersed in the surrounding tissue matrix. While these characteristics of the DA remain unchanged, it undergoes a series of drastic morphological changes^11^.

At 24 hpf, the embryonic heart starts beating and initiates blood circulation. From that moment on, DA rapidly increases its diameter from ~ 24 to ~ 32 µm at 42.5 hpf. Then at around 40 hpf, the aorta develops a periodic pattern of alternating thinner and thicker regions (see Fig. 1). Its appearance coincides with the initial extrusion events of future HSPCs. After peaking at 42.5 hpf, the DA diameter starts to decrease. At 65 hpf, the aorta recovers its original cylindrical shape and diameter^11^.

Interestingly, the HSPC extrusion rate peaks between 42.5 and 52 hpf precisely when the aorta diameter starts to decrease^11^. High-resolution fluorescent microscopy also allows following fates of individual cells and reveals details of the EHT at cellular level. An endothelial cell destined to become a hematopoietic one migrates toward the ventral part of the dorsal aorta (it is still unknown when precisely cell fate differentiation takes place). Once there, the cell drastically changes its morphology. Starting as a flat elongated cell with virtually equal apical and basal membranes areas (characteristic diameter ~ 10 µm) and submicron thickness, it undergoes a strong anteroposterior contraction and acquires a round plate-like shape, buckling outside of the dorsal aorta lumen toward the cardinal vein^11,13,34^. The process is finalized by the formation and closure of an actin ring around the emerging cell. The actin ring or actomyosin ring is the most archetypical structure of cytokinesis, which is an essential part of cell division. The ring contains actin filaments cross-linked by the motor protein Myosin II^51^. Neighboring endothelial cells also assist the process by accommodating their shape and ensuring aorta integrity to prevent blood hemorrhage.

## Results and discussion

### Continuous model of the dorsal aorta

Models based on the concept of energy minimization have found much success in describing self-organization processes, including morphogenesis^1,21,52,53^. Here, we develop a micromechanical model of the dorsal aorta. We define the elastic energy of the system and, importantly, we show how the regulation of mechanical properties and stress generation assists the molecular mechanism driving EHT.

As shown in Fig. 1, morphological changes of the DA arising during the EHT process are reminiscent of deformations observed in biological and non-biological systems with similar geometry. For example, analogous deformations occur in ordinary thin cylindrical shells under axial load^17,19^, swelling gels^54^, and tubular lipid membranes^25^. Despite the similarities between tubular lipid membranes (TLMs), whose stability was extensively studied in our previous works^25,27,55^, the DA has a number of features distinguishing it from lipid tubes.

A major difference is a nonzero shear modulus of the DA. Unlike lipid membranes with virtually non-existent shear modulus, zebrafish DA, composed of endothelial cells, resists all types of deformations (shear, bending, and stretching/compression) because of the rigidity of cytoskeleton of individual cells and cell to cell adhesion^7^. In mathematical terms, this means that the deformation energy stored in the system depends not only on the final shape of the tube but also on all the displacement field components (unlike TLM deformation energy, which is a function of radial displacements only). Thus, we model the DA as a two-dimensional solid cylindrical shell (such description of monolayer epithelium as a two-dimensional sheet is quite common^8,9,52,56^) and parametrize its surface as:

1 $$\begin{gathered} x^{\prime} = \left[ {\left( {R + {u_r}} \right)\cos \varphi - {u_\varphi }\sin \varphi } \right] \hfill \\ \;y^{\prime} = \left[ {\left( {R + {u_r}} \right)\sin \varphi + {u_\varphi }\cos \varphi } \right] \hfill \\ \;z^{\prime} = z + {u_z} \hfill \\ \end{gathered}$$

where *R* is the tube radius in relaxed state and ${\varvec{u}}=[{u}_{r},{u}_{\varphi },{u}_{z}]$ is a displacement field characterizing the DA surface in cylindrical coordinates. Since the DA has a nonzero shear modulus, the vector ${\varvec{u}}$ has both radial and tangential components.

Another essential feature of the DA is that it finds itself in a stressed state for most of the developmental process (like many other biological tissues in physiological conditions^57^). The stress should have both inhomogeneous and homogeneous components. The inhomogeneous contribution may be associated with the difference in the EC expansion/contraction rates and blood flow, whereas homogeneous one may originate from hydrostatic blood pressure and different growth rates of the aorta and surrounding tissues. We start with a simplified symmetric model accounting for only homogeneous stress and then discuss its possible modifications. Basing on the observed shape of the DA and its displacement relative to the surrounding tissues, we have already hypothesized that axial load induced by the tissue growth rate mismatch plays a crucial role in aorta morphogenesis^11^. Different growth rates of two adjacent tissues can lead to the situation when the faster growing tissue is under confinement, and the growth rate difference causes effective compression. Confined growth of tissues is actively studied and attributed to the formation of the shapes of different organs^20,22^. Here we assume that the dorsal aorta is subjected to the homogeneous stress ${\sigma }_{zz}$ along its axis together with hydrostatic blood pressure and write the energy of the DA as follows:

2 $${\Phi}={\int }_{S}\left[\frac{\lambda }{2}{\left({\varepsilon }_{ii}\right)}^{2}+\mu {\varepsilon }_{ik}^{2}+\frac{\kappa }{2}\left(\Delta {{H}^{^{\prime}}}\right)^{2}- {\sigma }_{zz}{\partial }_{z}{u}_{z}\right]dS-V\Delta P,$$

where ${\varepsilon }_{ik}$ is a two-dimensional nonlinear deformation tensor (please, see Supplementary materials Eq. S4), $\lambda$ and $\mu$ are two-dimensional analogs of Lame coefficients, $\kappa$ is a bending rigidity, $\Delta {H}^{^{\prime}}=({\partial }_{\varphi }^{2}{u}_{r}+{R}^{2}{\partial }_{z}^{2}{u}_{r}-{\partial }_{\varphi }{u}_{\varphi })/{R}^{2}$ is a renormalized average curvature variation^58^. The integration is performed over the DA surface *S*. *V* stands for the DA volume and $\Delta P$ stands for the pressure difference in the system (for more details see Supplementary materials Eq. S6).

Homogeneous mechanical stress induced by the external tissues and hydrostatic pressure (terms ${\sigma }_{zz}{\partial }_{z}{u}_{z}$ and $V\Delta P$) leads to a homogeneous deformation of the cylindrical shell, maintaining its symmetry. A total displacement field (caused by both thermal fluctuations and external forces) of the DA surface can be represented as:

3 $${\varvec{u}}={{\varvec{u}}}_{0}+{{\varvec{u}}}^{\prime},$$

where the homogeneous part is given as ${{\varvec{u}}}_{0}=\left[{\varepsilon }_{\varphi \varphi }^{0}R,0,{\varepsilon }_{zz}^{0}z\right]$. By substituting ${\varepsilon }_{\varphi \varphi }^{0}$ and ${\varepsilon }_{zz}^{0}$ in Eq. (2) and minimizing the equation with respect to these quantities, one can obtain that the field ${{\varvec{u}}}_{0}$ corresponds to the following linear deformations of the membrane:

4 $${\varepsilon }_{\varphi \varphi }^{0}=\frac{\Delta PR\left(\lambda +4\mu \right)-2\lambda {\sigma }_{zz}}{8\mu \left(\lambda +\mu \right)},{\varepsilon }_{zz}^{0}=\frac{2{\sigma }_{zz}\left(\lambda +2\mu \right)-\Delta PR\left(\lambda -2\mu \right)}{8\mu \left(\lambda +\mu \right)}$$

We assume that both control parameters $\Delta PR$ and ${\sigma }_{zz}$ are significantly smaller (in absolute value) than any of the Lame coefficients. Thus, we ensure that deformations (4) are small enough to be considered as perturbations of the initial state. Now we can find the free energy of the system $\Delta \Phi\left({{\varvec{u}}}^{\boldsymbol{^{\prime}}}\right)$, as a function of the additional virtual displacement ${{\varvec{u}}}^{\boldsymbol{^{\prime}}}$, taking stressed cylinder with energy $\Phi\left({{\varvec{u}}}_{0}\right)$ as a reference state:

5 $$\Delta \Phi\left({{\varvec{u}}}^{{^{\prime}}}\right)=\Phi\left({\varvec{u}}\right)-\Phi\left({{\varvec{u}}}_{0}\right).$$

After performing all the substitutions in Eq. (5), we use the relations ${\sigma }_{zz} ,\Delta PR\ll \lambda , \mu$ and linearize the model. Thus, we retain only terms of the first order in ${\sigma }_{zz}$ and $\Delta P$ and terms of the first and second orders in the components of the displacement field ${{\varvec{u}}}^{\boldsymbol{^{\prime}}}$ and its spatial derivatives:

6 $$ \begin{aligned}\Delta\Phi &={\Delta \Phi}^{\left(1\right)}+{\int }_{S}\left[\left(\frac{\lambda }{2}{\left({\varepsilon }_{ii}^{^{\prime}}\right)}^{2}+\mu \left({{\varepsilon }_{ik}^{^{\prime}}}^{2}\right)\right)+{\sigma }_{zz}{J}_{1}+R\Delta P{J}_{2}+{\frac{\kappa }{2}\left(\Delta {H}^{^{\prime}}\right)}^{2}\right]dS, \\ { \Delta \Phi}^{\left(1\right)}&=\frac{\Delta P}{2}{\int }_{S}{\partial }_{\varphi }{u{^{\prime}}}_{\varphi }dS, \\ {J}_{1}&=\frac{1}{2}{\left({\partial }_{z}{u{^{\prime}}}_{r}\right)}^{2}+\frac{1}{8}{\left({\partial }_{z}{u{^{\prime}}}_{\varphi} - \frac{1}{R}{\partial }_{\varphi}{u{^{\prime}}}_{z} \right)}^{2}, \\ {J}_{2}&=\frac{1}{2}{J}_{1}+\frac{1}{8}{\left({\partial }_{z}{u{^{\prime}}}_{\varphi }-\frac{1}{R}{\partial }_{\varphi }{u{^{\prime}}}_{z}\right)}^{2}+\frac{1}{2R}\left({\partial }_{z}{u{^{\prime}}}_{\varphi }{\partial }_{\varphi }{u{^{\prime}}}_{z}-{\partial }_{z}{u{^{\prime}}}_{z}{\partial }_{\varphi }{u{^{\prime}}}_{\varphi }\right)\\ &\quad+\frac{{\partial }_{\varphi }{u{^{\prime}}}_{r}}{{2R}^{2}}\left({\partial }_{\varphi }{u{^{\prime}}}_{r}-{u{^{\prime}}}_{\varphi }\right)-\frac{{{u}^{\prime}}_{r}}{2{R}^{2}}\left({{u}^{^{\prime}}}_{r}+{\partial }_{\varphi }{{u}^{^{\prime}}}_{\varphi }+{R\partial }_{z}{{u}^{^{\prime}}}_{z}\right)\end{aligned} $$

where ${\varepsilon }_{ik}^{^{\prime}}$ is the linearized deformation tensor^58^.

Before proceeding to the stability analysis, let us justify the assumed smallness of displacement fields ${\varvec{u}}$ and ${\varvec{u}}\boldsymbol{^{\prime}}$. Biological tissues under physiological conditions often exhibit nonlinear elastic properties, meaning that material constants should be redefined at every step of the deformational process. Such approach is often used to describe living tissues^57^ and is known as mechanics of incremental deformation^59^. In addition to elastic deformation, the morphogenesis of the DA involves genetically preprogrammed processes leading to the variation of the size and mechanical properties of the cells. We use an adiabatic approximation and assume that the system evolves through the sequence of equilibrium states^3,21^. This approach allows us to use our linearized model for the stability analysis at various steps of the process, even though DA dimensions change substantially during the considered period. Critical deformations, driving the instabilities can be considered as small perturbations with respect to the stretched but still highly symmetrical cylindrical state.

Providing the DA has the length *L* we expand the displacement field associated with these perturbations as:

7 $${u{^{\prime}}}_{j}\left(\varphi ,z\right)={\sum }_{n=-\infty }^{\infty }{\sum }_{m=-\infty }^{\infty }{{(A}_{n,m}^{j}e}^{i\left({k}_{m}z+n\varphi \right)}),$$

where ${k}_{m}=\frac{2\pi m}{L}$, *n* and *m* are wave numbers and ${A}_{n,m}^{j},(j=1..3$) are complex amplitudes of the field harmonics. After substitution of Eq. (7) in Eq. (6) and integration the linear term in Eq. (6) disappears to obtain:

8 $$\Delta \Phi=2\pi RL\sum_{n,m=-\infty }^{\infty }{M}_{n,m}^{i,j}{A}_{n,m}^{i}{A}_{n,m}^{{j}^{*}}$$

where the matrix ***M*** has the following form:

9 $$\left[\begin{array}{ccc}\frac{\lambda +2\mu }{{R}^{2}}+\frac{\kappa {X}^{2}}{{R}^{4}}+\frac{\Delta P(X+{n}^{2}-2)}{2R}+{\sigma }_{zz}{k}_{m}^{2}& -i\left(\frac{\kappa X}{{R}^{4}}+\frac{\lambda +2\mu }{{R}^{2}}\right)n& -i\left(\frac{\lambda -\Delta PR}{R}\right){k}_{m}\\ i\left(\frac{\kappa X}{{R}^{4}}+\frac{\lambda +2\mu }{{R}^{2}}\right)n& \frac{{R}^{2}\left(\lambda +2\mu \right)+\kappa }{{R}^{4}}{n}^{2}+\left(\mu +\frac{{\sigma }_{zz}}{4}+\frac{3\Delta PR}{8}\right){k}_{m}^{2}& \left(\frac{\lambda +\mu }{R}-\frac{{\sigma }_{zz}}{4R}-\frac{3\Delta P}{8}\right)n{k}_{m}\\ i\left(\frac{\lambda -\Delta PR}{R}\right){k}_{m}& \left(\frac{\lambda +\mu }{R}-\frac{{\sigma }_{zz}}{4R}-\frac{3\Delta P}{8}\right)n{k}_{m}& \left(\lambda +2\mu \right){k}_{m}^{2}+\left(\mu +\frac{{\sigma }_{zz}}{4}+\frac{3\Delta PR}{8}\right)\frac{{n}^{2}}{{R}^{2}}\end{array}\right]$$

$$X={R}^{2}{k}_{m}^{2}+{n}^{2}$$

The matrix ***M*** defines the shape of the DA. In the high symmetry phase, the matrix ***M*** should satisfy the inequality $\mathrm{Det}({\varvec{M}})>0$, which means that all modes of the displacement field ${{\varvec{u}}}^{\boldsymbol{^{\prime}}}$ vanish at equilibrium, and DA preserves the shape of a straight circular cylinder. Points of the parameter space $\langle \Delta PR,{\sigma }_{zz}\rangle$, which violate this inequality for specific *n* and *m* wave numbers, correspond to the adjacent phases with critical modes of the displacement field lowering DA symmetry. When building the model, we have not made any assumptions concerning symmetry of these phases; therefore, analyzing ***M*** is sufficient for finding all instabilities changing the cylindrical shape of the aorta. In the following sections, we examine critical modes of the DA and determine the stability region of the system.

### Critical modes with rotational symmetry

After the matrix ***M*** has been obtained, numerical stability analysis is relatively straightforward, providing material constants characterizing the system are known. Unfortunately, measuring their values in vivo is extremely challenging in zebrafish DA. Currently, we have no experimental data on material constants at any point in time relevant to EHT. Moreover, they are also likely to change throughout the whole developmental process. Therefore, an analytical study of the DA stability is currently the most rational option. We perform it below by introducing several assumptions that do not affect the generality of the result obtained.

We start with the case when the system preserves its rotational symmetry and assume *n* = *0*, which corresponds to so-called corrugation modes. Thus the matrix elements *M*_*12*_*,M*_*21*_*,M*_*23*_*,M*_*32*_ turn to zero and the equation $\mathrm{Det}({\varvec{M}})=0$ takes the following form:

10 $${M}_{11}{M}_{33}-{M}_{13}{M}_{31}=0$$

Since DA radius is much smaller than its length (*R* << *L*)*,* we can consider the wave vector ${k}_{m}$ as a variable with a continuous spectrum of values. Solving the Eq. (10) with respect to ${\sigma }_{zz}$ and minimizing the result with respect to ${k}_{m}$ (by its absolute value), we obtain the following expression for the critical wave vector:

11 $${{k}_{m}}^{2}=\sqrt{\frac{4\mu \left(\lambda +\mu \right)-\Delta PR(\Delta PR-\lambda +2\mu )}{\kappa \left(\lambda +2\mu \right){R}^{2}}}$$

As we shall demonstrate later, the critical radial corrugation mode with this wave vector is responsible for the destabilization of the DA. Using the relations ${\sigma }_{zz} ,\Delta PR\ll \lambda , \mu$, one can obtain a corresponding critical value of ${\sigma }_{zz}$:

12 $${\sigma }_{zz}^{corr}=-\frac{\Delta PR}{2}\left(1+2\left(2{\nu }_{2D}-1\right)\sqrt{\frac{\kappa }{{E}_{2D}{R}^{2}}}\right)-\frac{2\sqrt{\kappa {E}_{2D}}}{R}$$

where ${E}_{2D}=\frac{4\mu \left(\lambda +\mu \right)}{\lambda +2\mu }$ is a two-dimensional Young modulus and ${\nu }_{2D}=\frac{\lambda }{\lambda +2\mu }$ is a two-dimensional Poisson’s ratio. Since the thickness of the DA wall is small compared to the DA diameter, we can assume that $\kappa \ll {E}_{2D}{R}^{2}$, meaning the expression in the brackets is approximate to $1$. As Eq. 12 shows, negative longitudinal stress ${\sigma }_{zz}$, arising due to the difference in the growth rates of the aorta and surrounding tissues, may cause a mechanical instability, that leads to the formation of the corrugation pattern. On the other hand, the stretching stress term $-\frac{\Delta PR}{2}$, corresponding to blood pressure dilating the tube, tends to stabilize the system. It is also worth noting that, according to Eq. 12, two factors may contribute to the eventual loss of the DA stability. First, in Eq. 12, the increase of the aorta radius decreases the value of the second term associated with the rigidity of the system. Moreover, the dilatation may also weaken the hydrostatic pressure, thus, effectively decreasing the forces which stabilize the system. Simultaneously, during the development of the embryo, the growth rate mismatch leads to an increase in the negative longitudinal stress, which ultimately exceeds the stabilizing forces and causes the instability in the DA. Later in the text, we discuss in greater detail the development of the instability in vivo.

Equation (12) is reminiscent of the classical expression for the stability of the axially loaded cylindrical shell:

13 $${\sigma }_{zz}^{corr}=-\frac{2\sqrt{DEh}}{R}$$

where *h* stand for the wall thickness, *E* is Young modulus, and *D* is the cylindrical rigidity of the shell^17^.

### Instabilities violating rotational symmetry

In this section, we examine the stability of the system with respect to modes violating rotational symmetry. Euler buckling is a well-known instability arising in axially loaded tubes and rods leading to the bending of their main axis^18^; it has also been found in adult arteries^52,60,61^. The author of the work^61^ studied the buckling of an artery subjected to hydrostatic blood pressure and axial tension. According to their results, the artery may buckle even under stretching tension ${\sigma }_{zz}>0$, providing a sufficient pressure delating the tube $\Delta P>0$ is applied. The wave vector of this instability is reported to be ${k}_{m}=\pi /L$. Buckling instability with the same wave vector develops in axially loaded rigid rod with pinned ends^18^. As proven by experimental observations, this type of instability is not provoked in the DA during hematopoiesis because of the surrounding tissues^11,34^, like, for example, the rigid notochord, which is in close contact with the DA apical part. Nevertheless, since our model can be applied to other tubular structures, it is interesting to compare the stabilities of the buckling mode and the corrugation mode studied in the previous section. To do that, we solve the equation $\mathrm{Det}({\varvec{M}})=0$, taking the wave number *n* = *1*. In this case, the determinant of the matrix ***M***, Eq. (9) is an eighth-order polynomial with respect to the wave vector ${k}_{m}$, which has the following form:

14 $$D={a}_{1}{k}_{m}^{2}+{a}_{2}{k}_{m}^{4}+{a}_{3}{k}_{m}^{6}+{a}_{4}{k}_{m}^{8}$$

where ${k}_{m}=\frac{2\pi m}{L}$ and coefficients ${a}_{i}$ are functions of the control parameters $\Delta P$ and ${\sigma }_{zz}$. The determinant (14) does not contain any free terms and thus vanishes at ${k}_{m}=0$. This trivial solution represents the zero-energy Goldstone mode that corresponds to the tube translation as a whole and has no influence on the stability of the system. To calculate the critical value of stress ${\sigma }_{zz}$ leading to the instability that breaks the rotational symmetry of the system, we introduce a normalized wave vector $k={k}_{m}R$. Following the work^52^, we use the long tube approximation (*L* >> *R*) and assume that the normalized wave vector $k$ has a small yet finite value. In this case, the critical ${\sigma }_{zz}$ value can be estimated from the equation ${a}_{1}{k}^{2}+{a}_{2}{k}^{4}=0$. Taking into account the smallness of the wave vector ($k<<1$) and using the assumption $\Delta PR\ll \mu ,\lambda$, we obtain the following expression for the critical load:

15 $${\sigma }_{zz}^{buck}=-2\mu \frac{{\left(\lambda +\mu \right)R}^{2}+\kappa }{{\left(\lambda +2\mu \right)R}^{2}+\kappa }{k}^{2}$$

Now let us consider another type of instability invoked by the pressure difference $\Delta P$. This time we assume that after destabilization, the tube preserves its translational symmetry and thus substitute ${k}_{m}=0$ in the matrix ${\varvec{M}}$. If $\left|n\right|>1$, the following inequality governs the stability of the system:

16 $${M}_{11}{M}_{22}-{M}_{12}{M}_{21}>0$$

Modes with $\left|n\right|\le 1$ should be considered separately since translation and rotation of the tube as a whole break the inequality (16) but do not break the tube stability. Solving Eq. (16) with respect to $\Delta P$ and taking into account the relations $\kappa \ll \lambda {R}^{2},\mu {R}^{2}$, we obtain the expression for the critical value of the pressure difference:

17 $$\Delta P>-\frac{\left({n}^{2}-1\right)\kappa }{{R}^{3}}.$$

From the inequality (17), one can easily obtain that the instability is associated with a two-fold degenerate mode ${|n|=2,k}_{m}=0$ and occurs when the pressure difference reaches the critical value:

18 $$\Delta P=-\frac{3\kappa }{{R}^{3}}$$

According to this classic result^17^, the rigid cylindrical tube cross-section subjected to a sufficient hydrostatic compression takes an oval shape. Analogously to the previously discussed Euler instability, however, this transversal buckling is irrelevant for the DA, since the pressure difference caused by the blood flow is obviously positive. Observed ovality of the DA cross-section is due to an anisotropic compression applied by the muscles located on the sides of the aorta and not due to the transverse buckling with spontaneous symmetry breaking caused by the blood pressure^11^.

Since EHT process is associated with deformation and, importantly, buckling of future HSCPs, it is interesting to shortly discuss the stability of individual cell within the theoretical framework we introduced. Obviously, the stability region is different from that of the whole tube. To estimate it, we model the cell as an approximately flat square plate with sides of length *a* = *R*. Then the critical stress value is given by the following classic equation:

19 $${\sigma }_{zz}=-\kappa {k}^{2}$$

where $k={l}_{c}\pi /R$ and the coefficient ${l}_{c}$ depends on the type of boundary conditions. Based on the experimentally observed shape of the extruding cells and behavior of their borders during EHT^11,13,34^, we believe that pinned boundary conditions are the most suitable ones; for the cell with pinned borders ${l}_{c}=1$. Equation (19) can be obtained from the equation ${M}_{11}=0$ after performing the limiting transition from cylindrical to planar geometry, i.e., $R\to \infty$. Equation 19 shows that buckling instability can be initiated by the compressive stress or changes in the rigidity $\kappa$ of the extruding cell. The stress may be associated with passive processes such as inhomogeneous growth or active ones such as actomyosin contraction. This result will be used afterward.

### Stability domain of the cylindric phase

**Figure 2:**
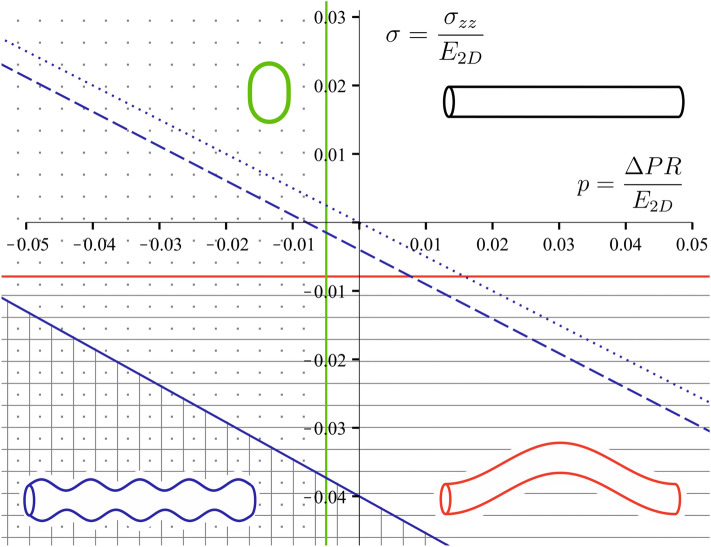
Stability domain of the tube with radius to length ratio $R/L=1/50$. High symmetry phase is located in the non-shaded area. The red line corresponds to the critical values of the axial stress ${\sigma }_{zz}$ leading to the Euler buckling instability. The green vertical line corresponds to the critical value of the pressure leading to the transversal buckling with wave numbers |*n|*= 2, *m* = 0 of the tube with bending rigidity $\gamma =1/25$. The set of blue lines corresponds to the critical values of the stress ${\sigma }_{zz}$ leading to the corrugation instability of tubes with various normalized bending rigidities: solid line $\gamma =1/25$, dashed line $\gamma =1/250$, dotted line $\gamma =0$.

Let us compare the tube stability with respect to the corrugation mode, the transversal buckling mode, and the long-wave buckling mode of the system; to do that we introduce small dimensionless parameters: the normalized pressure $p=\Delta PR/{E}_{2D}$, the normalized axial stress $\sigma ={\sigma }_{zz}/{{E}_{2D}}$, and the normalized bending rigidity $\gamma =\sqrt{\kappa /{E}_{2D}{R}^{2}}$. For the sake of clarity, we consider the tube made of an incompressible material, taking ${\nu }_{2D}\approx 1$ (unlike in 3D, where incompressible material corresponds to $\nu =1/2$). Then by keeping only the lowest non-vanishing terms in $\gamma$ and $\kappa$ we rewrite Eqs. (12), (15), and (18) as:

20 $${\sigma }^{corr}\approx -\left[\frac{p}{2}\left(1+2\gamma \right)+\gamma \right]$$

21 $${\sigma }^{buck}\approx -\frac{1}{2}{\left(\frac{2\pi R}{L}\right)}^{2}$$

22 $${p}^{trans}\approx -3{\gamma }^{2}$$

with ${\sigma }^{corr}, {\sigma }^{buck}$ being normalized axial stress values leading to the corrugation and buckling instabilities in the DA, accordingly, and ${p}^{trans}$ being normalized pressure leading to the transverse buckling of the DA cross-section. Equation (21) shows that the critical value of the axial stress ${\sigma }_{zz}$ leading to the Euler buckling instability is proportional to the *R/L* ratio squared, whereas the stability of the corrugation mode is independent of the relative length of the tube (see Eq. (20)). Figure 2 shows the stability domain of the system in the parameter space $\langle p,\sigma \rangle$ for the tube with radius to length ratio $R/L=1/50$, which agrees with the observations in zebrafish embryo. In the considered approximation, critical values of the control parameters corresponding to the Euler instability lie along the straight horizontal line (depicted in red). The corrugation mode loses stability along the straight line with a slope $\approx -\frac{1}{2}$, which crosses the y-axis at the point $\sigma =-\gamma$. And finally, vertical straight line $p=-3{\gamma }^{2}$ corresponds to the transversal buckling instability associated with the mode *|n|*= 2, *m* = 0.

### Contribution of surrounding tissues to the stability of the system

In the previous sections, we have analyzed how the material constants characterizing the tube influence the stability domain of the high-symmetry cylindrical phase. The influence of the surrounding tissues has only been considered indirectly by restricting the symmetry of the low-symmetry phase. In this section, we show that the stability of the DA strongly depends on the properties of the surrounding medium. The simplest way to consider an interaction between the tube and the outer tissues is to introduce the term ${\int }_{s}\frac{C}{2}{{u}_{r}}^{2}dS$ to the energy (6), where $C$ is a phenomenological radial pinning coefficient^60,62^. Since in general for tubular shells immersed into a rigid matrix, the tangential pinning is much weaker than the radial one^60,62,63^, for the sake of simplicity, in the energy (6), we keep only the term proportional to the square of radial displacement. Let us note that according to the experimental data between 30 and 60 hpf, individual cells migrate along the DA surface, which also justifies why we discard the tangential pinning in the energy (6). This modification of the energy leads to the emergence of the single additional term $C$ in the element *M*_*11*_ of the matrix (9).

Consequently, the inequality (17) describing the stability of the system with respect to transversal buckling modes is modified to:

23 $$\Delta P>-\frac{CR}{\left({n}^{2}-1\right)}-\frac{\left({n}^{2}-1\right)\kappa }{{R}^{3}}$$

If the pinning effect is relatively weak $C{R}^{4}\le \kappa$, the transversal buckling instability is associated with the mode |*n|*= 2, *m* = 0, like in the free tube. However, when the interaction with the medium is significant $C{R}^{4}/\kappa \gg 1$, the tube loses stability with respect to modes with higher wave numbers |*n|*> 2. The expression for the critical ${\sigma }_{zz}$ value (12) corresponding to the tube corrugation is also modified and takes the following form:

24 $${\sigma }_{zz}=-\frac{\Delta PR}{2}\left(1+2\left(2\nu -1\right)\sqrt{\frac{\kappa }{{E}_{2D}^{{^{\prime}}}{R}^{2}}}\right)-\frac{2\sqrt{\kappa {E}_{2D}^{{^{\prime}}}}}{R}$$

where ${E{^{\prime}}}_{2D}={E}_{2D}+C{R}^{2}$ is an effective Young modulus. Thus, the previous estimation of the critical stress value leading to the corrugation remains relevant, the only difference being an effective increase of the Young modulus of the tube, caused by the introduction of the pinning contribution into the model. On the contrary, the buckling modes with ($n=\pm 1,k=\pm 2\pi /L$) become non-critical because of an additional constant term that appears in the polynomial (14).

An analogous result was obtained by the authors of the work^52^ where a similar system was studied. The authors showed that in an artery surrounded by a rigid tissue matrix, instability is associated with corrugation modes (in their paper, this type of instability is referred to as “varicose instability”) and not with classical Euler buckling modes, like in the work^61^. Despite some similar results regarding the influence of the environment on the possible critical modes of the tube, our work has several significant differences from the paper^52^. First of all, in Ref.^52^, artery is not subjected to a real axial longitudinal mechanical stress. Instead, a homogeneous isotropic tension is introduced; this tension is analogous to the liquid–liquid interface energy in the theory of lipid membranes. The authors of the work^52^ justify the use of such, unconventional for solids, type of energy by the complex structure of arteries. They model arteries as elastic tubes covered by a monolayer of epithelial cells from inside. According to this reference, epithelial cell division and growth in confinement caused by surrounding tissue results in an analog of an isotropic interface energy. While conceptually this idea is analogous to the compression by growth rate mismatch, we believe that mathematical description, developed in Ref.^52^, is inapplicable for the DA description. As the experimental data shows^11–13,34^, the ECs deform inhomogeneously, which is crucial for the EHT. Thus, a concept of conventional anisotropic stress typical of solids should be used.

### Bridging theory and experiment

Now, let us analyze the development of the zebrafish dorsal aorta between 25 and 65 hpf—period relevant for the definitive wave of hematopoiesis—within the model framework, and validate our model by comparing its predictions to new and published experimental data. While focusing on the changes in the mechanical properties of the system accompanying hematopoiesis, we also briefly discuss microenvironmental cues leading to these changes. We consider DA morphogenesis as a movement of the system in the parameter space, $\langle p,\sigma \rangle$, of the normalized pressure difference and the normalized longitudinal stress respectively. As it was previously mentioned, we assume that the system passes through a succession of equilibrium states.

**Figure 3:**
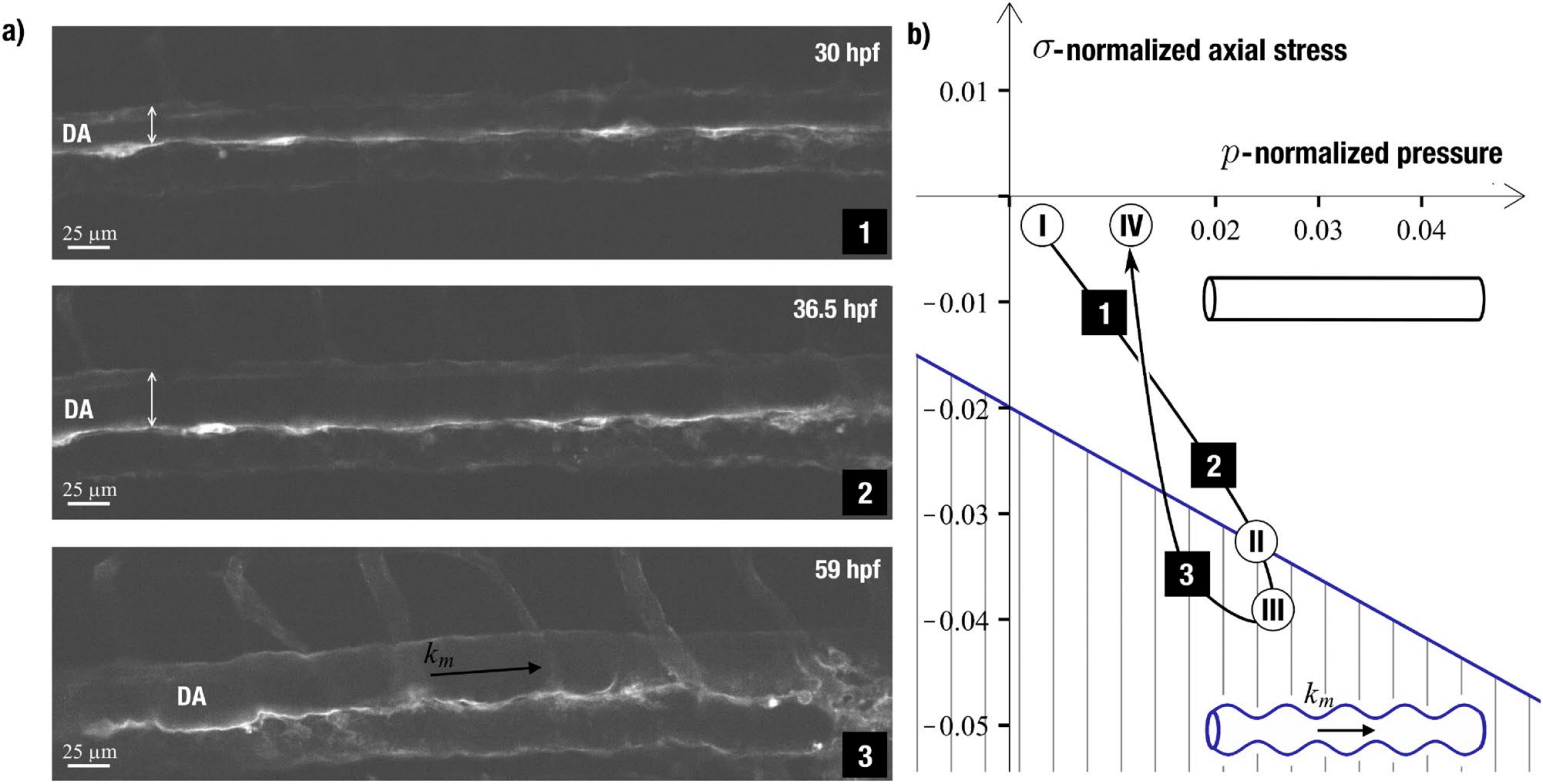
Live imaging of a zebrafish dorsal aorta morphogenesis from 30 to 60 hpf. (**a**) Pictures extracted from the live imaging of the *Tg(kdrl:caax-mCherry)* zebrafish embryo segments shows the DA morphological changes at 30 hpf, 36.5 hpf and 59 hpf. Arabic numerals conform to the **b** panel. (**b**) Trajectory describing the developmental process in the parameter space $\langle p,\sigma \rangle ($i.e., normalized pressure difference and normalized longitudinal stress). Arabic numerals correspond to the images in the **a** panel. Roman numerals correspond to the DA shape evolution stages: (I) initiation of the DA expansion; (II) emergence of the periodic pattern of thinner and thicker regions; (III) peaking of the DA radius; (IV) restoration of the initial shape of a straight cylinder. Diagram corresponds to the tube with $\gamma =1/50$. Double-headed arrows show DA diameter variation. Black arrow shows instability wave vector. Scale bar: 25 µm. See also Supplementary Videos S1 to S4.

In order to reinforce experimental data on the values of material constants characterizing the system, we perform a zeroth-order estimate. This parameter provides a better context for how the model reacts to changes in these values in physiologically relevant ranges and to give an idea of how material constants can be deduced for future experimental studies. The parameter $\gamma =\sqrt{\kappa /{E}_{2D}{R}^{2}}$ can be estimated within the Foppl von Karman theory for thin membranes^18^. According to this theory, the bending rigidity $\kappa$ is expressed as $\frac{{E}_{2D}{h}^{2}}{12\left(1-{{\nu }_{2D}}^{2}\right)}$, where *h* is the membrane thickness^62^. We choose the DA radius to be $R\approx 14 \mu m$, which corresponds to the period when it loses stability with respect to corrugation mode (see, Fig. 3). Assuming wall thickness is $h\approx 500\mathrm{ nm}$, and 2D Poisson’s ratio is ${\nu }_{2D}\approx 0.9$, we obtain $\gamma \approx 1/50$. Choosing such an estimate of 2D Poisson’s ratio, we follow the approximation that epithelial cells are nearly incompressible^64^. And finally, the pinning coefficient that describes interaction with surrounding tissues only renormalizes the Young modulus when dealing with corrugation mode, making the system more robust to bending (see Eq. 20). In the phase diagram (Figs. 2 & 3), even a two- or three-fold increase of the estimated normalized bending rigidity only slightly changes the slope of the blue line, which separates corrugated and cylindrical phases, and lowers the line down. The phase diagram does not change qualitatively; the system can switch between phases while staying in the same quadrant of the parameter space, meaning that positive hydrostatic blood pressure and compressive longitudinal stress can drive the process.

The trajectory describing the system development can be subdivided into the following steps (Fig. 3):

1. At around 24 hpf right before the start of a heartbeat, the pressure difference in the system should remain negligible, and the dorsal aorta has a shape of a cylinder with a diameter of 24 ± 0.8 µm^11^. With the initiation of the blood flow and the rise of the hydrostatic blood pressure, the DA diameter starts to increase with the expansion of the EC apical/basal membranes. The increase of the aorta diameter and pressure difference contributes to the increase of the normalized pressure difference $p$. While the difference in the growth rates of aorta and surrounding tissues may cause axial compression, it is still insufficient to destabilize the system and cause symmetry breaking deformations.
2. At around 40 hpf, the aorta develops the periodic pattern with sinusoidal modulation of DA diameter. This means that the axial stress, caused by the interaction with the surrounding tissues, eventually overpowers the stabilizing effect of the hydrostatic pressure and provokes corrugation instability with the vector *k*_*m*_ (depicted by the black arrow in Fig. 3).
3. The DA average diameter peaks at approximately 42 hpf, reaching 32 ± 0.9 µm. From that moment on, the EC expansion can no longer compensate for DA surface area decrease due to the extrusion of the future HSPCs. This process gradually relieves the longitudinal stress in the DA walls, which corresponds to the decrease of $\sigma$. The aorta diameter reduction corresponds to the decrease of the parameter $p$, even at constant blood pressure.
4. Eventually, the decrease of $\sigma$ brings the system back to the high symmetry phase. At approximately 65 hpf, the aorta restores its initial shape and diameter (those at 24 hpf) and HSPCs production stops.

Comparing experimental data to modeling results, one can notice that observed deformations are inhomogeneous with respect to the angular coordinate (see Fig. 3a). Multiple studies report that corrugation has the maximum amplitude in the dorsal part of the aorta and is absent in the ventral one, which is only natural considering that DA is confined by the rigid notochord at the top and by the muscle tissue at the sides. However, when performing stability analysis, we assume that elastic constants characterizing the aorta and its surroundings do not depend on the azimuthal angle. This assumption is crucial for finding an analytical solution to the problem. We indirectly account for the inhomogeneities by selecting instabilities relevant for the zebrafish DA and extrapolate the results on the experimental data. While inhomogeneous rigidity of surrounding tissues indeed plays an important role in the process, allowing cells to extrude from the DA in the vicinity of the cardinal vein so they can enter the blood flow, qualitatively results of our analysis still hold, and the predicted succession of shape phase transitions agrees with experimental observations.

To better understand how mechanical forces orchestrate the HSPCs production, let us compare DA shape evolution in wild-type (WT) and mutant embryos. In Refs.^12,45^, it has been shown that dorsal aorta of silent-heart (SH) mutants with no blood flow and mutants with 50%-reduced blood flow are severely flattened and have decreased cross-section area with respect to WT embryos. Reduction of the average DA radius has also been observed in mutant embryos with disrupted mechanosensing^12^. Based on these results and ability of the ECs to sense shear stress^48,65,66^, we speculate that in WT embryos, blood flow-induced shear stress activates genetic mechanisms leading to the expansion of the individual cells, thus overall aorta radius increase. Importantly, aorta of the SH mutants is thinner than that of mutants with 50%-reduced blood flow and with disrupted mechanosensing. This allows us to assume that, while the shear stress mainly plays a signaling role, the hydrostatic blood pressure directly stretches the tube and helps it preserve a more circular cross-section. Translational symmetry of the DA also points toward the secondary role of the blood flow-induced shear forces in deforming the aorta directly. If the DA shape had strongly depended on the shear stress value, then the DA radius, initiation of the corrugation instability, and cell extrusion should have been highly inhomogeneous along the DA length, which is not observed in WT embryos. Corrugation instability signaling that the longitudinal stress has reached a critical value appears in all the previously considered cases, regardless of the blood flow, which is also in accordance with our model; the lack of hydrostatic blood pressure means lowered stability of the DA to the longitudinal stress originating from the growth rate mismatch (see Eq. 12). In the developed theoretical framework, the development of mutant embryos is described by trajectories with a steeper slope in the $\langle p,\sigma \rangle$ space since the decrease of both the blood pressure and the DA radius leads to the decrease of the normalized pressure $p$. The steeper the trajectory, the faster it reaches the corrugation phase (see Fig. S2), which is in accordance with experimental data^12,45^. However, it is important to note that mutants rarely survive the whole period of 24–65 hpf, meaning that in reality, corresponding trajectories would be incomplete.

Now let us focus on the cellular level and discuss the roles of the blood flow and mechanosensing in the EHT. It has recently been shown^12,45^ that both the blood flow inhibition and abnormal mechanosensing lead to premature initiation of the extrusion process. Moreover, the process itself is disrupted in the mutants. Cells are extruded not only outward but also inward the aorta. Upon the extrusion, they have an abnormal ovoid shape instead of a cup-like one. Many cells burst when leaving the aorta. Because of premature extrusion, ECs are not subjected long enough to the molecular and mechanical environmental cues, including shear stress, necessary to specify and maintain HSPC identity^12,44,67^, which hinders the HSPC production.

Disruption of actomyosin machinery normal functioning affects cell extrusion similarly to the absence of blood flow^11,12,45^; the cells buckle abnormally (inside and outside of aorta), do not undergo anterior–posterior constriction like in WT embryos, many of them are extruded only partially and burst. Thus, even though some sort of cell buckling occurs in mutant embryos, actomyosin ring formation around the cells, which are ready to leave the aorta, and its subsequent contraction are essential for the successful extrusion.

Based on the abovementioned results, one can readily make two assumptions. First, gene expression controlling assembly and contraction of the actomyosin ring is probably activated by the shear stress. That is why blocking the blood flow itself, suppressing the ability of ECs to sense it, and disrupting normal actomyosin machinery, all have very similar effects on the EHT process.

Second, buckling of the individual cells toward the sub-aortic space preceding the cell extrusion is another mechanical instability driving the HSPC production. The instability is activated by the plane stress in the aorta walls and not by the blood pressure. The authors of Ref.^13^ expressed an analogous idea by stating that both the deployment of pushing forces by the adjoining endothelial cells and the actin ring contraction drive the EHT process.

Figure 3a shows that wave vector ${k}_{m}$ of the DA corrugation instability (Eq. (11)) is larger than characteristic cell size and, thus, the wave vector $k$ of the buckling instability (Eq. (19)) in an individual cell. One can conclude that buckling mode in most ECs comprising the aorta is more stable than the critical corrugation mode in the DA. Nevertheless, some cells, particularly ones undergoing EHT, do buckle even in embryos with abnormal actomyosin machinery. Thus, it is natural to assume that the differentiation starts prior to the cell extrusion and changes mechanical properties of future HSPCs, making them less stable to buckling than other cells in DA walls. However, when and how ECs ‘decide’ to become HSPCs is yet to be discovered.

While there are some discrepancies in experimental data, results of several studies suggest^11,12,34^ a correlation between formation of corrugation pattern and initiation/peak in EHT events. Considering this fact, the formation of the corrugation pattern may be viewed as an indication of the stress reaching a critical value that is sufficient for the extrusions of future HSPCs. However, a connection between these two events requires further experimental studies to be confirmed. Such an interpretation agrees with the blood flow reduction leading to premature cell extrusion. As we have shown, blood pressure stabilizes the system serving as a counteracting force to the compression induced by the growth rate mismatch between the aorta and the surrounding tissues. Thus, in the absence of blood flow, longitudinal stress in the aorta walls reaches the critical value earlier, leading to both the formation of the aorta corrugation pattern and the buckling of the individual cells.

## Conclusion

We have studied the evolution and instabilities of the zebrafish dorsal aorta shape during the definitive wave of hematopoiesis. A new micromechanical model of the DA has been developed. Based on our analytical results, new 4D confocal microscopy data, and previously published experimental results, we argue that conversion of ECs, comprising the aorta, into HSPCs is accompanied by two mechanical instabilities arising in the system due to the compressive longitudinal stress in the DA wall. The first instability leads to the appearance of the periodic pattern of alternating thinner and thicker regions; the second one leads to the buckling of the individual ECs destined to become HSPCs. We show that, while it is known that the blood flow-induced shear stress serves as a signal controlling gene machinery, it is hydrostatic blood pressure that directly stabilizes and stretches the dorsal aorta, affecting its shape during hematopoiesis. Thus, mechanical forces not only serve as signals promoting and synchronizing HSPCs production but also to assist the process by activating shape instabilities directly.

## Methods

### Zebrafish husbandry

*Tg(kdrl:Has.HRAS-mCherry)* (here cited as *kdrl:caax-mCherry)*^68^ were maintained, crossed, raised and staged as described previously^69,70^. All animal experiments described in the present study were conducted at the University of Montpellier according to European Union guidelines for handling of laboratory animals (http://ec.europa.eu/environment/chemicals/lab_animals/home_en.htm) and were approved by the Direction Sanitaire et Vétérinaire de l'Hérault and Comité d'Ethique pour l'Expérimentation Animale under reference CEEA-LR-13007.

### Microscopy

Time-lapse imaging was performed using Nikon Spinning Disk at 20X magnification as described in previously^71^. Embryos were anesthetised with tricaine (0.016%) and mounted on a glass cover dish with 0.7% low melting agarose and covered with standard E3 medium supplemented with tricaine and 1-phenyl-2-thiourea (PTU) (0.003%) to prevent pigment formation. Temperature was maintained at 28 °C by placing the dish in a temperature-control chamber during time-lapse acquisitions. Images were analysed using ImageJ.

## Supplementary Information

Supplementary Video 1.

Supplementary Video 2.

Supplementary Video 3.

Supplementary Video 4.

Supplementary Information 1.
