# Author Correction: Modeling and live imaging of mechanical instabilities in the zebrafish aorta during hematopoiesis

**DOI:** 10.1038/s41598-021-95765-2

**Published:** 2021-08-05

**Authors:** Dmitrii Chalin, Charlotte Bureau, Andrea Parmeggiani, Sergei Rochal, Karima Kissa, Ivan Golushko

**Affiliations:** 1 Research and Education Center “Materials”, Don State Technical University, 1 Gagarin Square, Rostov-on-Don, 344003 Russia; 2 LPHI, University of Montpellier, CNRS, INSERM, Montpellier, France; 3 Laboratoire Charles Coulomb, University of Montpellier, CNRS, Montpellier, France; 4 Faculty of Physics, Southern Federal University, Zorge 5, Rostov-on-Don, 344090 Russian Federation

Correction to: *Scientific Reports* 10.1038/s41598-021-88667-w, published online 29 April 2021

In the original version of this Article Dmitrii Chalin was incorrectly affiliated with ‘Faculty of Physics, Southern Federal University, Zorge 5, Rostov-on-Don, 344090, Russian Federation’. The correct affiliation is listed below.

Research and Education Center “Materials”, Don State Technical University, 1 Gagarin Square, Rostov-on-Don, 344000, Russia.

The original Article and accompanying Supplementary Information file have been corrected.

