# Supplementary material for: Modeling and live imaging of mechanical instabilities in the zebrafish aorta during hematopoiesis
Source: Sci Rep. 2021 Apr 29;11:9316. doi: 10.1038/s41598-021-88667-w (PMC8085226; doi:10.1038/s41598-021-88667-w)
Supplement: Supplementary file 5 — Supplementary Information 1. [file 41598_2021_88667_MOESM5_ESM.docx]

**Modeling and live imaging of mechanical instabilities in the zebrafish aorta during hematopoiesis**

Dmitrii Chalin^1^, Charlotte Bureau^2^, Andrea Parmeggiani^2,3^, Sergei Rochal^4^, Karima Kissa^2^*, Ivan Golushko^1^*

^1^Research and Education Center "Materials", Don State Technical University, 1 Gagarin Square, Rostov-on-Don 344003, Russia

^2^LPHI, University of Montpellier, CNRS, INSERM, Montpellier, France

^3^Laboratoire Charles Coulomb, University of Montpellier, CNRS, Montpellier, France

^4^Faculty of Physics, Southern Federal University, Zorge 5, Rostov-on-Don, 344090, Russian Federation

*corresponding authors

# Supplementary materials

# Nonlinear strain tensor

To describe large-scale deformations in the dorsal aorta (DA), we use the following nonlinear strain tensor ^1,2^

| $\boldsymbol{\varepsilon}=\sqrt{\boldsymbol{g}}-\boldsymbol{\delta}$ | (S1) |
| --- | --- |

where the components of the metric tensor $\boldsymbol{g}$ are expressed as $g_{ij}=\frac{\partial\boldsymbol{r}^{\boldsymbol{'}}}{\partial w_{i}}\boldsymbol{\cdot}\frac{\partial\boldsymbol{r}^{\boldsymbol{'}}}{\partial w_{j}}$, vector $\boldsymbol{w=[}R\varphi,z\boldsymbol{]}$ parameterizes the surface of the cylindrical shell, and $\boldsymbol{\delta}$ is the identity matrix. By using such definition of the metric tensor, we provide that the components $g_{ij}$ are dimensionless. Thus, we can rewrite Eq. (S1) as

| $\boldsymbol{\varepsilon}=\sqrt{\boldsymbol{\delta}\boldsymbol{+}\Delta\boldsymbol{g}}-\boldsymbol{\delta}$ | (S2) |
| --- | --- |

where $\Delta\boldsymbol{g}$ is the part of the metric tensor associated with the deformations in the aorta. Provided $\Delta g_{ij}\ll1$, we can expand Eq. (S2) in series up to the second-order terms in ${\Delta g}_{ij}$:

| $\varepsilon_{ij}=\frac{{\Delta g}_{ij}}{2}-\frac{{\Delta g}_{ik}{\Delta g}_{kj}}{8}$ | (S3) |
| --- | --- |

After calculating the metric tensor of the strained surface in the explicit form, one can obtain the following expressions for its components (S3):

| $\varepsilon_{\varphi\varphi}=\frac{u_{r}+\partial_{\varphi}u_{\varphi}}{R}+\frac{\left( \partial_{\varphi}u_{z} \right)^{2}+\left( \partial_{\varphi}u_{r}-u_{\varphi} \right)^{2}}{2R^{2}}-\frac{\left( R\partial_{z}u_{\varphi}+\partial_{\varphi}u_{z} \right)^{2}}{8R^{2}}$  $\varepsilon_{\varphi z}=\frac{R\partial_{\varphi}u_{z}+\partial_{z}u_{\varphi}}{2R}+\frac{\partial_{z}u_{r}\left( \partial_{\varphi}u_{r}-u_{\varphi} \right)}{4R}+\frac{\left( R\partial_{z}u_{\varphi}-\partial_{\varphi}u_{z} \right)\left( u_{r}+u_{\varphi}\partial_{\varphi}-R\partial_{z}u_{z} \right)}{4R^{2}}$  $\varepsilon_{zz}=\partial_{z}u_{z}+\frac{\left( \partial_{z}u_{\varphi} \right)^{2}+\left( \partial_{z}u_{r} \right)^{2}}{2}-\frac{\left( {R\partial}_{z}u_{\varphi}+\partial_{\varphi}u_{z} \right)^{2}}{{8R}^{2}}$ | (S4) |
| --- | --- |

# Tubular membrane volume

Using the following expression one can find the DA volume $V$:

| $V=\int d^{3}\mathbf{R}^{\mathrm{in}}=\int_{0}^{1} \int_{0}^{2\pi} \int_{0}^{L} \left\vert\frac{\partial\left( x,y,z \right)}{\partial\left( \alpha,\varphi,z \right)} \right\vert d\alpha d\varphi dz$ | (S5) |
| --- | --- |

here $\mathbf{R}^{\mathrm{in}}=(\alpha x,\alpha y,z)$ is a set of points enclosed inside the cylinder, and the expression $\frac{\partial\left( x,y,z \right)}{\partial\left( \alpha,\varphi,z \right)}$ is a Jacobian of the transformation to a new coordinate system. After integrating over $\alpha$ and transitioning to an integration over the DA surface, we obtain the following expression for the deformed tube volume:

| $V=R\int_{0}^{1} \int_{0}^{2\pi} \int_{0}^{L} \left\vert\frac{\partial\left( x,y,z \right)}{\partial\left( \alpha,\varphi,z \right)} \right\vert d\alpha d\varphi dz=\int_{S} \left[ u_{r}\left( 1+\partial_{z}u_{z}+\frac{u_{r}}{2R}+\frac{\partial_{\varphi}u_{\varphi}}{2R}+\frac{u_{r}\cdot\partial_{z}u_{z}}{2R}+\frac{\partial_{\varphi}u_{\varphi}{\cdot\partial}_{z}u_{z}}{2R} \right)+\frac{u_{\varphi}}{2R}\left( u_{\varphi}-\partial_{\varphi}u_{r}-\partial_{z}u_{z}\cdot\partial_{\varphi}u_{r}+u_{\varphi}\cdot\partial_{z}u_{z} \right)+\frac{1}{2}{\partial_{\varphi}u}_{\varphi}\left( 1+\partial_{z}u_{z} \right)+\frac{1}{2}R{\partial_{z}u}_{z}-\frac{1}{2}{\partial_{\varphi}u}_{z}\cdot\left( {\partial_{z}u}_{\varphi}-\frac{u_{\varphi}\cdot\partial_{z}u_{r}}{R}+\frac{u_{r}\cdot\partial_{z}u_{\varphi}}{R} \right)+\frac{1}{2}R \right]dS$ | (S6) |
| --- | --- |

Now, let us consider the volume of the cylinder described by the deformation field (3). Since the components of the homogeneous displacement field $\boldsymbol{u}_{\mathbf{0}}$ (see Eq. (4)) are linear in stress $\sigma_{zz}$ and pressure $\Delta P$, all the terms in the expression $-V\Delta P$ containing $\sigma_{zz}$ and/or $\Delta P$ are at least of the second order in these quantities. In the present work, we consider the stability of the system within the linearized theory framework and retain only the terms, that are linear in $\sigma_{zz}$ and $\Delta P$. Thus in the expression for the strained DA energy (6), volume variation turns out to be independent of the initial equilibrium displacement field $\boldsymbol{u}_{\boldsymbol{0}}$. In fact, the expression for the volume variation in Eq. (6) becomes identical to Eq. (S6) after replacing $\boldsymbol{u}$ with $\boldsymbol{u}^{\boldsymbol{'}}$ in Eq. (S6) and excluding the third-order terms in displacements and their derivatives (since we develop a harmonic theory).

# Supplementary figures

**
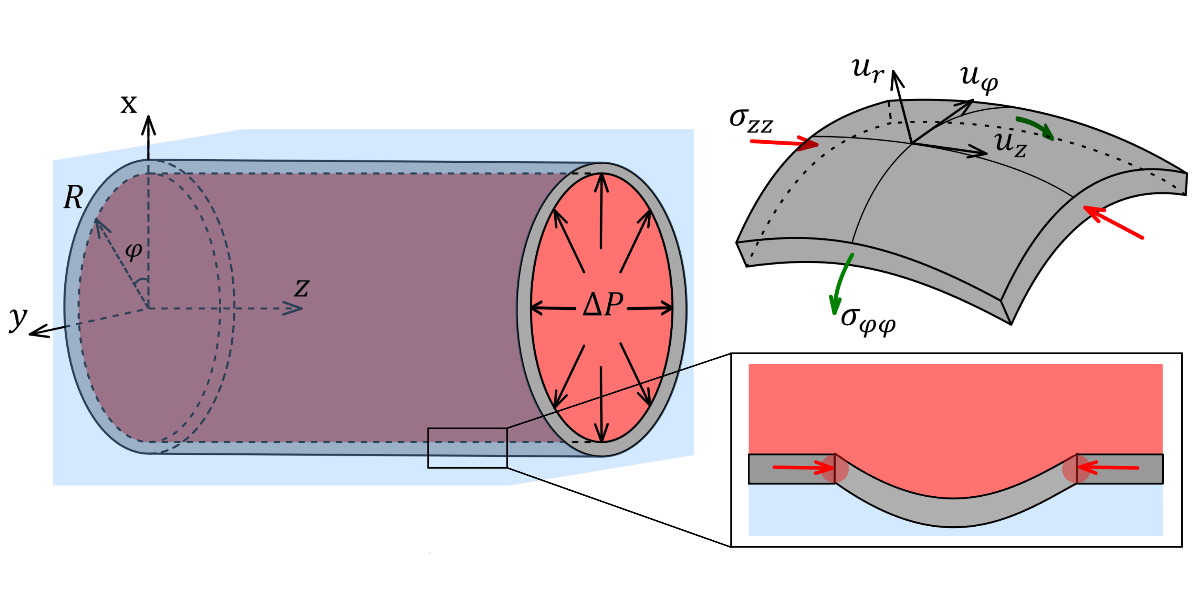
**

**Supplementary Figure S1.** Parametrization of the dorsal aorta surface. Colored arrows represent stress components associated with blood pressure and growth rate difference between aorta and surrounding tissue matrix. Insert shows buckling instability of individual cell. Green circles represent ‘pinning points’. Wall thickness is added for the sake of clarity (we model aorta as a 2D manifold in 3D space).

**
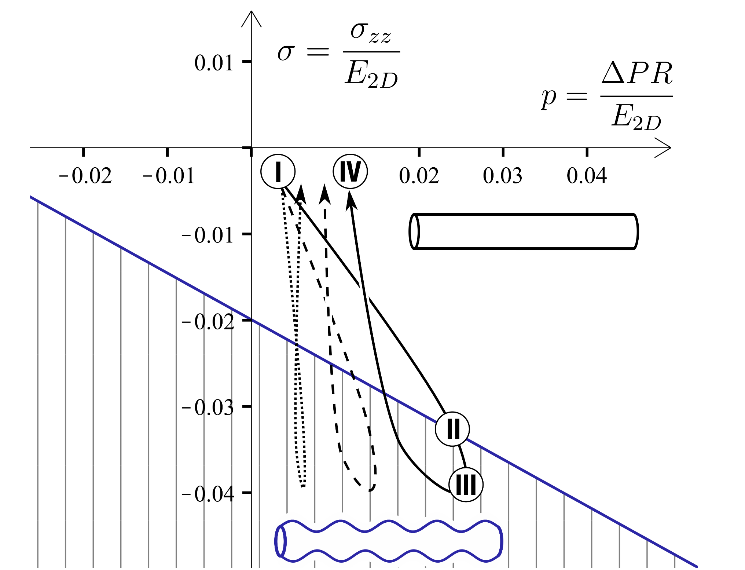
**

**Supplementary Figure S2.** Approximate trajectories describing the development of the DA embryos in the parameter space $\left\langle p,\sigma\right\rangle($i.e., normalized pressure difference and normalized longitudinal stress). Solid black line: wild-type embryo; roman numerals correspond to the DA shape evolution stages: I) initiation of the DA expansion; II) emergence of the periodic pattern of thinner and thicker regions; III) peaking of the DA radius; IV) restoration of the initial shape of a straight cylinder. Dashed black line: embryos with 50%-reduced blood flow. Dotted black line: silent-heart embryos. Solid blue line corresponds to the corrugation instability. Diagram corresponds to the tube with $\gamma=1/50$.

# Supplementary videos

**Supplementary Video S1.** Time lapse confocal fluorescence imaging of a zebrafish embryo from 30 to 60 hpf. The movie complements Figure 3 and demonstrates the development of the dorsal aorta in Tg(kdrl:caax-mCherry) embryo from 30 to 60 hpf. This movie shows a maximum projection from 20 z-stacks spaced by 3µm. Scale bar: 25 µm.

**Supplementary Video S2.** The movie shows a z-stack of aorta spaced by 3 µm. It allows for the visualization of aorta morphology at 30 hpf. Scale bar: 25 µm.

**Supplementary Video S3**. The movie shows successive z-stack of aorta spaced by 3 µm. It allows for the visualization of aorta morphology at 36.5hpf. Scale bar: 25 µm.

**Supplementary Video S4.** The movie shows successive z-stack of aorta spaced by 3 µm. It allows for the visualization of aorta morphology at 59 hpf.

The videos S2, S3, S4 were used to produce the still images in Figure 3 a.

# References

1. Chalin, D., Avramenko, M., Parmeggiani, A. & Rochal, S. Low-frequency phonon dynamics and related thermal properties of axially stressed single-walled carbon nanotubes. *J. Phys. Condens. Matter* **31**, (2019).

2. Biot, M. A. *Mechanics of incremental deformations*. (1965).
